# Identification of Biomarkers Related to Prognosis of Bladder Transitional Cell Carcinoma

**DOI:** 10.3389/fgene.2021.682237

**Published:** 2021-08-09

**Authors:** Zhihua Liu, Lina Xu, Youcheng Lin, Huaishan Hong, Yongbao Wei, Liefu Ye, Xiang Wu

**Affiliations:** ^1^Provincial Clinical Medical College of Fujian Medical University, Fuzhou, China; ^2^Department of Urology, South Blanch of Fujian Provincial Hospital, Fuzhou, China; ^3^Department of Urology, Fujian Provincial Hospital, Fuzhou, China

**Keywords:** bladder transitional cell carcinoma, biomarker, bioinformatics, TCGA, The Cancer Genome Atlas

## Abstract

Bladder transitional cell carcinoma (BTCC) is highly fatal and generally has a poor prognosis. To improve the prognosis of patients with BTCC, it is particularly important to identify biomarkers related to the prognosis. In this study, differentially expressed messenger RNAs were obtained by analyzing relevant data of BTCC from The Cancer Genome Atlas (TCGA) and Gene Expression Omnibus (GEO) databases. Next, hub genes that were suitable for correlation analysis with prognosis were determined through constructing a protein–protein interaction (PPI) network of differentially expressed genes and screening of major modules in the network. Finally, survival analysis of these hub genes found that three of them (*CCNB1*, *ASPM*, and *ACTC1*) were conspicuously related to the prognosis of patients with BTCC (*p* < 0.05). By combining the clinical features of BTCC and the expression levels of the three genes, univariate Cox and multivariate Cox regression analyses were performed and denoted that *CCNB1* could be used as an independent prognostic factor for BTCC. This study provided potential biomarkers for the prognosis of BTCC as well as a theoretical basis for subsequent prognosis-related research.

## Introduction

Bladder cancer (BC) is one of the most common and deadly diseases in the world, with approximately 430,000 new cases and more than 165,000 related deaths each year (Torre et al., 2015), making it the ninth largest malignant tumor worldwide. BC is a highly heterogeneous disease with different histological subtypes and prognoses, in which bladder transitional cell carcinoma (BTCC) is the major histopathological form, accounting for 90% of all BC cases (Siegel et al., 2019). Most BTCCs exhibit high tumor invasiveness. These malignancies originate from bladder mucosa and gradually invade the lamina propria of the urinary bladder. They sequentially enter the muscularis propria, fat tissue around the bladder, and adjacent pelvic structures. Meanwhile, lymph node metastasis occurs in high probability with malignant progression (Stein et al., 2001). Radical cystectomy is recognized as the standard treatment for this highly invasive BC, whereas data demonstrate that the 3-year survival rate of this treatment is only about 50% (Wang et al., 2017). Although surgical treatments have improved in recent years and certain types of new drugs have been released, about 50% of patients still suffer cancer metastasis or recurrence within 2 years after diagnosis (Sternberg et al., 2013). The American Joint Committee on Cancer (AJCC) staging system, as a conventional postoperative evaluation approach, is often used to predict the postoperative prognosis of BTCC patients (Wang and McKenney, 2019). However, studies manifested that the AJCC staging system is still deficient in predicting the prognosis of BTCC patients (Mackey et al., 1998; Dong et al., 2018). Recent studies assessed the mRNA and protein expression of tumor-related genes by immunocytochemistry, qPCR, Western blot, etc., thereby identifying biomarkers related to the prognosis of BTCC patients (Mackey et al., 1998; Li et al., 2018). Nevertheless, research in this area is still insufficient. Therefore, there is an urgent need to conduct deeper exploration of the pathogenesis of BTCC and find biomarkers related to the prognosis of BTCC to improve the prognosis of patients and alleviate the suffering of patients.

The screening of cancer-related biomarkers is an indispensable direction in cancer-related research. For example, in the research on renal cell carcinoma (RCC), it was found through The Cancer Genome Atlas (TCGA) and Oncomine databases that *GPX1* is highly expressed in RCC, and through Kaplan–Meier curve analysis that high *GPX1* levels predict a shorter overall survival (OS) time, indicating that *GPX1* has potential to be a biomarker for the diagnosis and prognosis of RCC patients (Cheng et al., 2019). In a study of pancreatic cancer, the expression of thymidylate synthetase (*TYMS*) in pancreatic cancer tissue and normal tissue is compared based on TCGA database, with its diagnostic value being explored through receiver operating characteristic (ROC) curve analysis, and Cox analysis revealed that the high expression of *TYMS* is related to poor OS and recurrence-free survival (RFS) (Fu et al., 2019). Thus, identification of cancer-related biomarkers can achieve early diagnosis of cancer patients and enable them to undergo corresponding treatment in time, so that they can have a good chance to realize disease-free survival (DFS), which is meaningful for improving the prognosis of patients.

The construction of protein–protein interaction (PPI) network is key to the screening process of cancer-related biomarkers. For example, Luo et al. (2019) imported the differentially downregulated genes of BC onto the STRING online database, constructed a PPI network, and then used MCODE for module analysis to identify important modules and seven important genes in the PPI network. Song et al. (2020) established a PPI network based on the differentially expressed genes (DEGs) related to hepatocellular carcinoma (HCC) in three data sets in Gene Expression Omnibus (GEO) database and screened the hub genes related to HCC for correlation analysis with prognosis. Wang et al. (2020) constructed a PPI network to obtain the hub genes of oral squamous cell carcinoma, analyzed the corresponding OS information of patients based on TCGA database, and finally screened out genes that are closely related to the survival rate of oral cancer patients. Therefore, cancer-associated hub genes can be accurately screened out through the construction of the PPI network.

Based on mining the corresponding data of BTCC in public databases, this study analyzed the DEGs in BTCC through bioinformatic methods to find notable prognosis-related biomarkers and attempted to provide a theoretical basis for the prognosis-related research on BTCC.

## Materials and Methods

### Data Downloading and Identification of Differentially Expressed Genes

The Cancer Genome Atlas Urothelial Bladder Carcinoma (TCGA-BLCA) gene expression data (tumor: *n* = 408; normal: *n* = 19) and corresponding clinical data (Supplementary Table 1) were downloaded from TCGA database^1^ in March 2020. GSE13507 chip data were obtained from GEO database^2^, including 165 primary BC samples, 23 recurrent non-muscle-invasive bladder cancer (NMIBC) samples, and 67 normal bladder mucosa tissue samples. Differential analysis was conducted on TCGA-BLCA data using the “edgeR” package, with | logFC| > 1 and false discovery rate (FDR) < 0.05 as thresholds for the selection of differentially expressed mRNAs (DEmRNAs). While for data from GSE13507, differential analysis was performed using the “limma” package, and the primary tumor samples and the recurrent tumor samples were, respectively, analyzed under the screening threshold of | logFC| > 1 and FDR < 0.05. Finally, the upregulated DEGs and downregulated DEGs in both data sets were intersected to obtain significant DEGs.

### Enrichment Analyses

To clarify the biological functions and pathways where the DEGs we identified were involved, the Metascape tool^3^ was used to perform Gene Ontology (GO) annotation and Kyoto Encyclopedia of Genes and Genomes (KEGG) pathway enrichment analyses. The Metascape is a website that integrates multiple authoritative data resources and can provide us with comprehensive and detailed information for each gene *via* performing pathway enrichment and biological process annotation. The results of GO analysis can show the biological functions of DEGs, including biological processes, cellular components, and molecular functions, while the results of KEGG pathway enrichment analysis can present the pathways involved in metabolism and signal transduction where DEGs were significantly enriched.

### Construction of Protein–Protein Interaction Network and Screening of Subnetworks

The DEGs were imported onto the online database STRING^4^ of known and predicted PPIs, and confidence score ≥ 0.4 was used as the cutoff value to construct a PPI network. The MCODE plug-in in Cytoscape (version 3.6.0) software was used to perform clustering analysis on genes in the PPI network (Degree cutoff = 2, Node Score cutoff = 0.2, K-Core = 2) for searching subnetworks and seed genes. Finally, ClueGo^5^, a plug-in that creates and visualizes a functionally grouped network of terms/pathways, was used to carry out a functional enrichment analysis on genes of the identified subnetworks.

### Identification and Verification of Hub Genes

The MCODE_Score top 30 genes and seed genes (Bader and Hogue, 2003) in the subnetworks identified by ClueGO functional enrichment analysis were selected for survival analysis based on patients’ OS and progression-free survival (PFS). OS is defined as the time from the diagnosis or treatment of cancer to the death of cancer patients (for any reason); PFS is defined as the time from the diagnosis or treatment of cancer to the progression of the tumor or death of cancer patients (for any reason). The data in TCGA-BLCA data set were grouped according to the median gene expression in all tumor samples. Then, log-rank test was used to compare the differences in OS and PFS between high- and low-expression groups, and finally, hub genes that were pronouncedly related to the prognosis of BTCC were obtained. Afterward, analysis of variance (ANOVA) was employed to compare the differences in the expression of the hub genes among normal samples, primary tumor samples, and recurrent tumor samples in GSE13507 data set. Besides, *t*-test was conducted on the GEPIA online website^6^ to analyze the difference in the expression of the hub genes between tumor tissue and normal tissue in TCGA database.

Finally, combined with BTCC clinical information and expression data of the hub genes in TCGA-BLCA data set, univariate Cox regression analysis was used to obtain factors notably related to prognosis, while multivariate Cox regression analysis was performed to analyze the obtained factors to determine whether these hub genes could be used as independent prognostic biomarkers.

## Results

### Analysis Results of Differentially Expressed Genes

**FIGURE 1:**
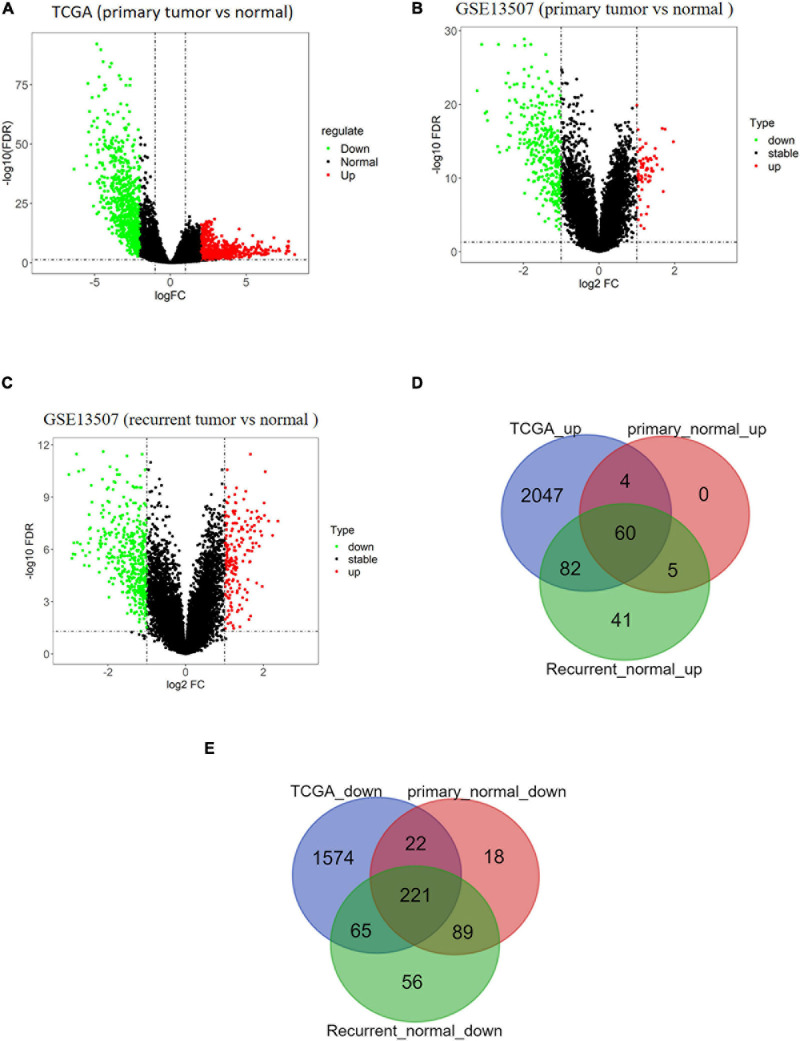
Analysis results of differentially expressed genes (DEGs) based on public databases. **(A)** Volcano map of DEGs of tumor/normal samples in The Cancer Genome Atlas (TCGA) data set, in which green dots represent downregulated genes and red dots represent upregulated genes. **(B)** Volcano map of DEGs of primary tumor/normal samples in GSE13507 data set, in which green dots represent downregulated genes and red dots represent upregulated genes. **(C)** Volcano map of DEGs of recurrent tumor/normal samples in GSE13507 data set, in which green dots represent downregulated genes and red dots represent upregulated genes. **(D)** Venn diagram of upregulated genes of TCGA data set and GSE13507 data set. The blue circle represents DEGs from TCGA, and red and green circles represent DEGs from GSE13507. **(E)** Venn diagram of downregulated genes of TCGA data set and GSE13507 data set. The blue circle represents DEGs from TCGA, and red and green circles represent DEGs from GSE13507.

BTCC-related data were downloaded from TCGA and GEO databases. A total of 2,193 upregulated genes and 1,882 downregulated genes in tumor samples were obtained from TCGA database (Figure 1A). Moreover, 69 upregulated genes and 350 downregulated genes in primary tumor samples (Figure 1B) and 188 upregulated genes and 431 downregulated genes in recurrent tumor samples were obtained from the GSE13507 data set (Figure 1C). Venn diagram illustrated that there were a total of 281 overlapping DEGs including 60 upregulated DEGs (Figure 1D) and 221 downregulated DEGs (Figure 1E).

### Functional Enrichment and Pathway Enrichment

**FIGURE 2:**
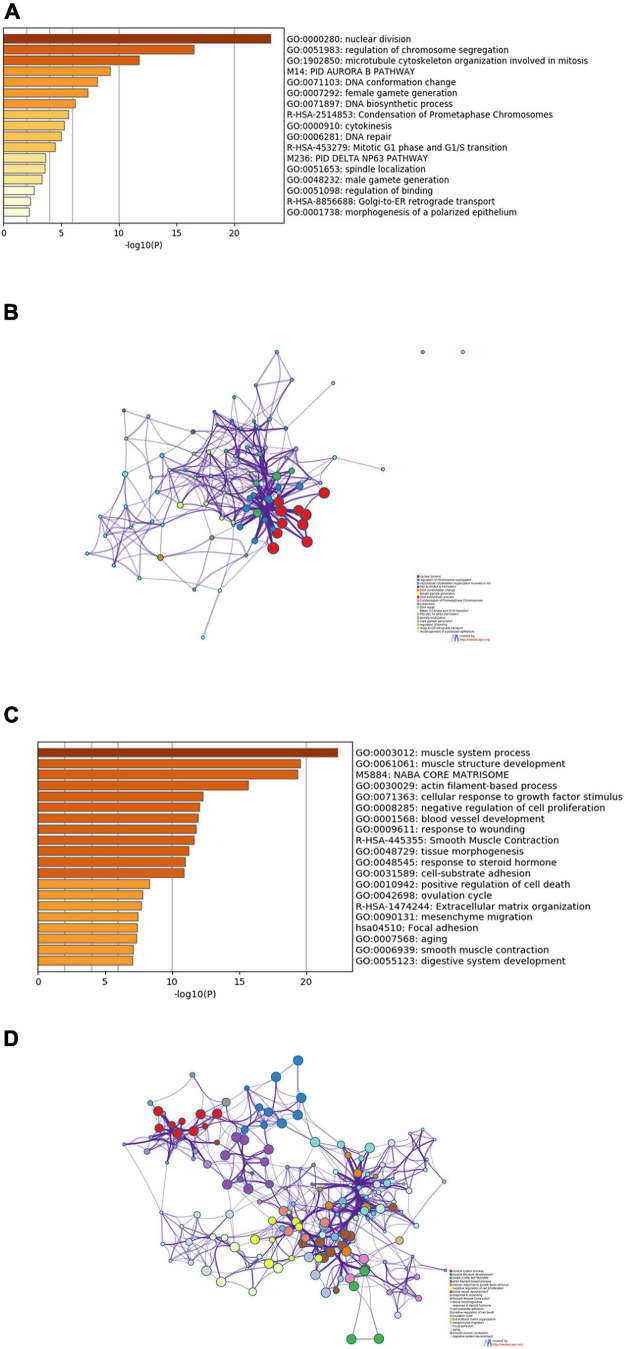
Results of functional and pathway enrichment analyses. **(A)** Bar graph of the top 20 enriched terms of upregulated genes. **(B)** Interaction network of upregulated genes, containing 20 sets of enrichment results. **(C)** Bar graph of the top 20 enriched terms of downregulated genes. **(D)** Interaction network of downregulated genes, containing 20 sets of enrichment results.

To explore the biological functions of the DEGs shared by TCGA-BLCA and GSE13507 data sets, Metascape was used to perform enrichment analysis on upregulated and downregulated DEGs and the first 20 enriched results were displayed, respectively. As shown in Figure 2A, these upregulated genes were dramatically enriched in biological functions such as nuclear division, regulation of chromosome segregation, and mitosis, as well as pathways such as PID AURORA B PATHWAY, Condensation of Prometaphase Chromosomes, and Mitotic G1 phase and G1/S transition. Figure 2B was a network showing the correlation between gene clusters enriched in the above biological functions and pathways.

Downregulated genes were enriched in biological functions such as muscle system process and muscle structure development, and were also markedly enriched in pathways such as NABA CORE MATRISOME and Smooth Muscle Contraction (Figure 2C). The downregulated gene network was presented in Figure 2D, in which genes in the same cluster were more closely related.

### Protein–Protein Interaction Network and Results of Module Analysis

**FIGURE 3:**
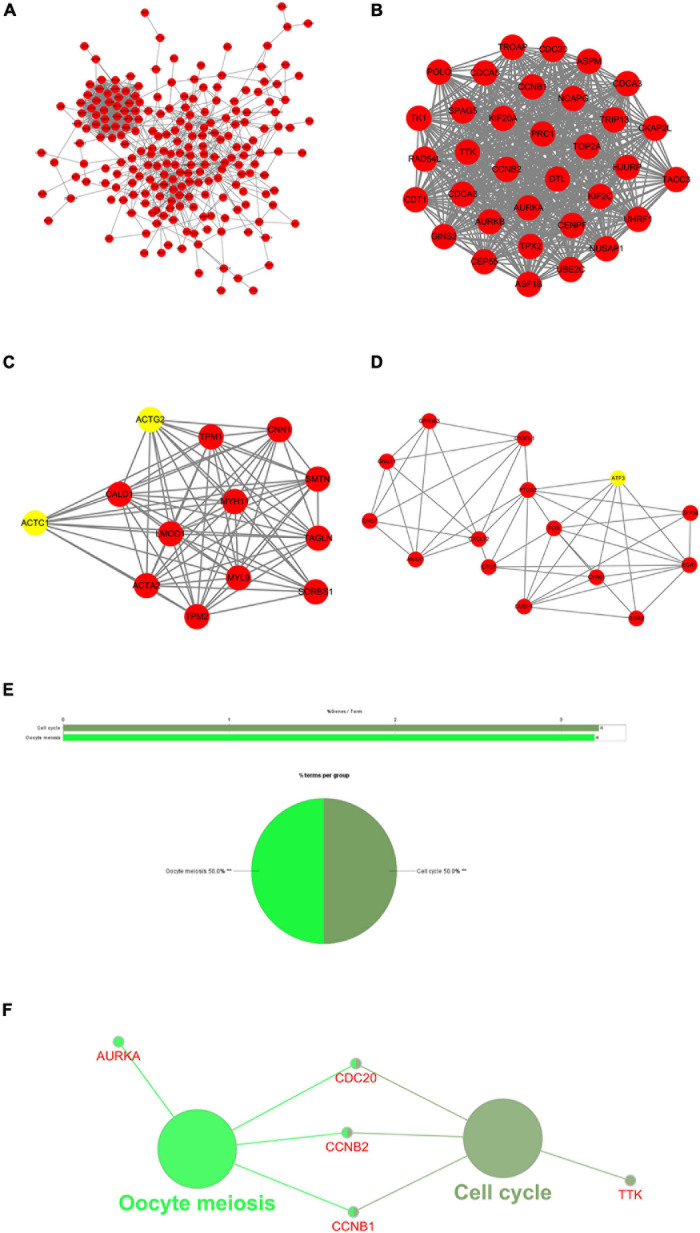
Protein–protein interaction (PPI) network and results of module analysis. **(A)** The PPI network of common differentially expressed genes (DEGs) in GSE13507 and The Cancer Genome Atlas Urothelial Bladder Carcinoma (TCGA-BLCA). **(B)** Module 1, MCODE score = 33.03. **(C)** Module 2, MCODE score = 12.167. **(D)** Module 3, MCODE score = 6.429 (yellow nodes are seed genes in the network; MCODE score refers to the score of each module to evaluate the degree of clustering). **(E)** Overview of the enrichment analysis of Module 1. **(F)** A functionally grouped network of terms, in which node size indicates the richness of the term, and the functionally related genes partially overlap. **represents *P* < 0.01.

To explore the association between proteins of DEGs shared by TCGA-BLCA and GSE13507 data sets, a PPI network of the DEGs was constructed containing 182 nodes and 1,130 relation pairs and visualized through Cytoscape (Figure 3A). Three subnetworks (Module 1, Module 2, and Module 3) (Figures 3B–D) and the seed genes (*ACTC1, ACTG2, ATF3*) in Module 2 and Module 3 were screened out by MCODE plug-in. Finally, ClueGO was used to perform functional enrichment analysis for the genes in subnetworks, and it was found that genes of Module 1 were enriched in cell cycle and oocyte meiosis (Figures 3E,F), whereas there were no significantly enriched pathways observed for the genes of Module 2 and Module 3.

### Significance of Hub Genes in the Prognosis of Patients With Bladder Transitional Cell Carcinoma

**FIGURE 4:**
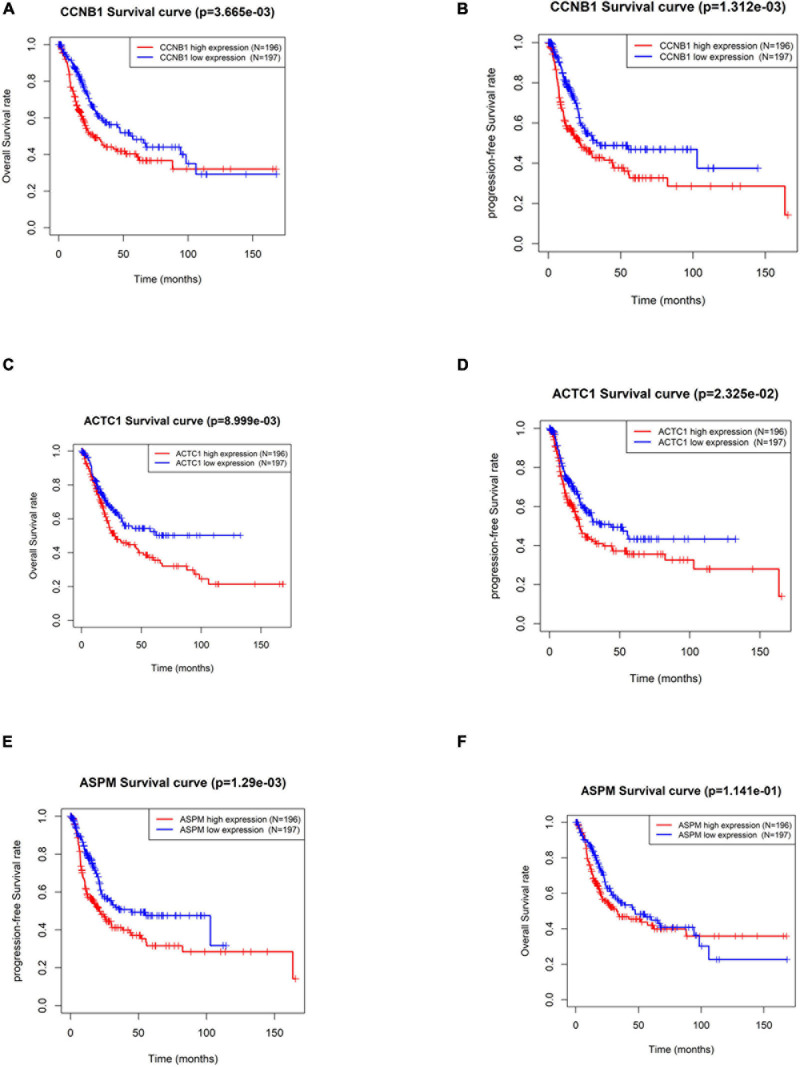
Significance of hub genes in the prognosis of patients with bladder transitional cell carcinoma (BTCC). **(A,B)** Overall survival (OS) and progression-free survival (PFS) analysis of patients based on high and low expression of CCNB1, respectively. **(C,D)** OS and PFS analysis of patients based on high and low expression of ACTC1, respectively. **(E,F)** OS and PFS analysis of patients based on high and low expression of ASPM, respectively. The red line represents high-expression group, and the blue line represents low-expression group.

By screening the subnetworks, the MCODE score of each gene in the subnetworks was obtained. In order to further explore their ability in predicting prognosis, a total of 33 hub genes including the top 30 genes of MCODE score in Module 1 (Supplementary Table 2) and seed genes in Module 2 and Module 3 were selected for subsequent analysis. In TCGA-BLCA data set, the median of gene expression in all tumor samples was used as the cutoff value for grouping. The results of Kaplan–Meier survival analysis (Supplementary Figure 1) exhibited that the OS rate and PFS rate of patients with high expression of Cyclin B1 (*CCNB1*) and Actin Alpha Cardiac Muscle 1 (*ACTC1*) were remarkably lower (*p* < 0.05) (Figures 4A–D). Furthermore, patients with high expression of Assembly Factor for Spindle Microtubules (*ASPM*) had a dramatically lower PFS rate (*p* < 0.05), but there was no significant difference in OS (Figures 4E,F). Overall, the three hub genes including *CCNB1*, *ASPM*, and *ACTC1* had good prognostic value in BTCC.

### Determination of Independent Prognostic Markers of Bladder Transitional Cell Carcinoma

**FIGURE 5:**
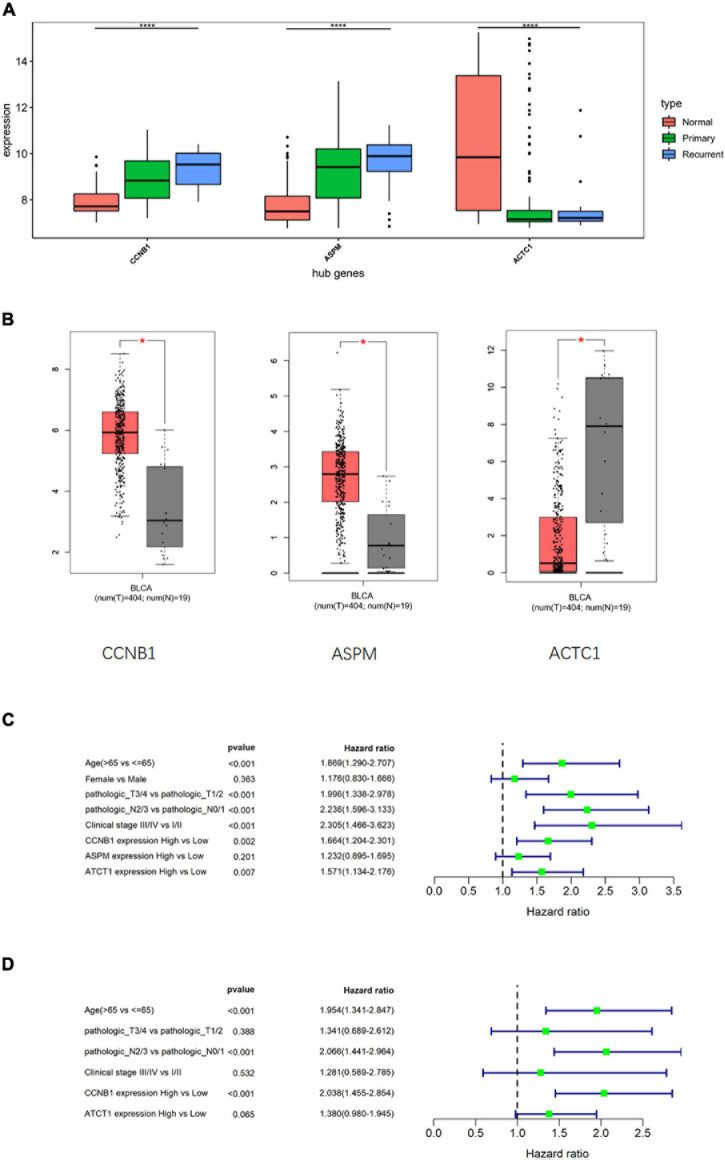
Cox analysis compares the diagnostic potential of hub genes and clinical features in bladder transitional cell carcinoma (BTCC). **(A)** Analysis of hub gene expression in GSE13507 data set, in which the red box represents the normal group, the green box represents the primary tumor group, and the blue box represents the recurrent tumor group. The horizontal line in the box represents the median; the upper and lower edges of the box represent the upper and lower quartiles; the upper and lower edges of the whisker represent the maximum and minimum observed values; the points beyond the upper and lower edges of the whisker represent outliers. The difference is verified by ANOVA. **(B)** Analysis of hub gene expression in The Cancer Genome Atlas Urothelial Bladder Carcinoma (TCGA-BLCA) data set, in which the red box represents the tumor group, and the gray box represents the normal group. The horizontal line in the box represents the median; the upper and lower edges of the box represent the upper and lower quartiles; the upper and lower edges of the whisker represent the maximum and minimum observed values; the points beyond the upper and lower edges of the whisker represent outliers. The difference is verified by *t*-test. **(C)** Univariate Cox regression analysis was performed on clinical features and hub gene expression in TCGA-BLCA data set. **(D)** Multivariate Cox regression analysis was conducted for the clinical features and hub genes that were notably related to survival in TCGA-BLCA data set; *p < 0.05, ****p < 0.0001.

To clarify the differences in the expression of hub genes in tumor tissue and normal tissue, the expression of the three hub genes was verified in GSE13507 and TCGA-BLCA data sets, respectively, and it was found that *CCNB1* and *ASPM* were highly expressed in tumor tissue (Figures 5A,B). Then, the relationship between cancer prognosis and expression of the hub genes or clinical features (age, gender, pathologic_T, pathologic_N, clinical_stage) was further analyzed in TCGA-BLCA data set to determine the prognostic value of the hub genes. Univariate Cox regression analysis depending on patients’ OS manifested that the difference in the expression of *CCNB1* or *ACTC1* was significantly related to the prognosis (*p* < 0.05) (Figure 5C). Multivariate Cox regression analysis was performed on variates that were conspicuously related to the prognosis as revealed by univariate analysis, and the results indicated that the expression of *CCNB1* was still remarkably related to OS (*p* < 0.001) (Figure 5D). Together, these results indicated that *CCNB1* could be used as an independent prognostic biomarker.

## Discussion

BTCC, also known as urothelial carcinoma of the bladder, is the most common type of BC (Siegel et al., 2019). For patients with BTCC, the main treatments are cystectomy and adjuvant chemotherapy, but the survival rate of patients markedly reduces when patients are likely to have tumor metastasis (Alfred Witjes et al., 2017). Therefore, neither surgical resection nor other adjuvant treatments can cure BTCC, and due to the efficacy of chemotherapy being limited by drug toxicity, drug resistance, and adverse reactions, the prognoses of patients are generally poor. Therefore, novel studies on biomarkers that are conspicuously associated with prognoses of patients have much clinical significance.

To explore the prognosis-related biomarkers of BTCC, a study conducted a differential analysis of the mRNA expression data of BC in TCGA database and then used least absolute shrinkage and selection operator (LASSO) Cox regression model to identify three N6-methyladenosine (m6A) RNA methylation regulators (Chen et al., 2019). Cao et al. (2020) also established a LASSO Cox regression model of epithelial–mesenchymal transition (EMT)-related genes through analysis of TCGA and GEO data and determined that EMT-related genes, pathologic_N stage, and age are independent factors for predicting the survival of patients with BTCC. While these studies propose several independent prognostic factors for BTCC, there are still some limitations. To make the results more objective and comprehensive, this study performed a differential analysis on the data from TCGA and the primary and recurrence tumor data in GEO database and then took an intersection to obtain 60 common upregulated DEGs and 221 downregulated DEGs. The GO and KEGG enrichment analyses revealed that these DEGs were prominently enriched in biological functions and pathways involved in nuclear division and the muscle system.

Next, in order to obtain hub genes related to BTCC prognosis, a PPI network was constructed using DEGs, and the subnetworks with better clustering were screened out, in which seed genes were used as hub genes for further analysis. In total, this study obtained 33 hub genes. Then, survival analysis was carried out to explore the relationship between the hub genes and the prognosis of patients with BTCC, with both OS and PFS taken into account. The results indicated that *ASPM, ACTC1*, and *CCNB1* were markedly correlated with the survival of patients with BTCC. Previous studies showed that *ASPM* (Kouprina et al., 2005), *ACTC1* (Jiang et al., 2020), and *CCNB1* (Deng et al., 2019) are related to cancer prognosis.

In this study, to determine whether these three genes can be used as independent prognostic biomarkers for BTCC, univariate and multivariate Cox regression analyses were conducted for the genes and clinicopathological features. Results showed that the hazard ratios of *CCNB1* in univariate and multivariate Cox regression analyses were both higher than 1 with the *p*-value lower than 0.05, indicating that *CCNB1* could be considered an effective and independent prognosis biomarker for BTCC to some extent. *CCNB1*, also known as *CyclinB1*, belongs to the family of highly conserved cyclins and is significantly overexpressed in various cancers (Miyazaki and Arai, 2007). *CCNB1* is proven to be a prognosis-related biomarker in a variety of cancers, including BC. For instance, four genes (*BUB1B*, *BUB1*, *TTK*, and *CCNB1*) that are pronouncedly upregulated in ovarian cancer and indicate dismal prognosis were disclosed through bioinformatic methods, and they can be used as potential therapeutic targets for patients with ovarian cancer (Feng et al., 2019). In breast cancer, *CCNB1* has a prominent predictive potential for distant metastasis-free survival, DFS, RFS, and OS of breast cancer patients, and it can be a biomarker of prognosis of breast cancer (Ding et al., 2014). Through the analysis of GEO data related to non-small-cell lung cancer (NSCLC), Ni et al. (2018) found six hub genes related to the pathogenesis and prognosis of NSCLC, including *CCNB1*. In view of the above findings, *CCNB1* can be used as a prognosis-related biomarker for a variety of cancers, and assuredly, there is no lack of related research on *CCNB1* as a prognosis-related biomarker for BC. For example, Gao et al. (2018) found that the expression level of *CCNB1* was correlated with the prognosis of patients with BC through bioinformatic analysis. These findings demonstrate that *CCNB1* as a prognosis-related biomarker has broad application prospects and great clinical significance and prove that the results of this study could provide potential biomarkers for the prognosis of BTCC.

Viewed in total, this study found a biomarker that could be used as an independent prognostic factor for BTCC. However, the study was conducted based on public databases and did not include experimental verification. Notwithstanding these limitations, the study can still provide a theoretical basis for further study of prognosis-related biomarkers of BTCC.

## Data Availability Statement

The original contributions presented in the study are included in the article/Supplementary Material, further inquiries can be directed to the corresponding author/s.

## Author Contributions

ZL and LX contributed to the study design. YL conducted the literature search. HH acquired the data. YL wrote the article. LY performed data analysis and drafted. XW revised the article. All authors gave the final approval of the version to be submitted.

## Conflict of Interest

The authors declare that the research was conducted in the absence of any commercial or financial relationships that could be construed as a potential conflict of interest.

## Publisher’s Note

All claims expressed in this article are solely those of the authors and do not necessarily represent those of their affiliated organizations, or those of the publisher, the editors and the reviewers. Any product that may be evaluated in this article, or claim that may be made by its manufacturer, is not guaranteed or endorsed by the publisher.

## Footnotes

1
https://portal.gdc.cancer.gov/

2
https://www.ncbi.nlm.nih.gov/geo/query/

3
https://metascape.org/gp/index.html#/main/step1

4
https://string-db.org/

5
http://apps.cytoscape.org/apps/cluego

6
http://gepia.cancer-pku.cn/

## Supplementary Material

Supplementary Figure 1 Kaplan-Meier survival analysis the for 30 hub genes.

Supplementary Table 1 Clinical data of TCGA-BLCA.

Supplementary Table 2 Hub genes.
