# Supplementary figures and images for: Identification of Biomarkers Related to Prognosis of Bladder Transitional Cell Carcinoma
Source: Front Genet. 2021 Aug 9;12:682237. doi: 10.3389/fgene.2021.682237 (PMC8381732; doi:10.3389/fgene.2021.682237)

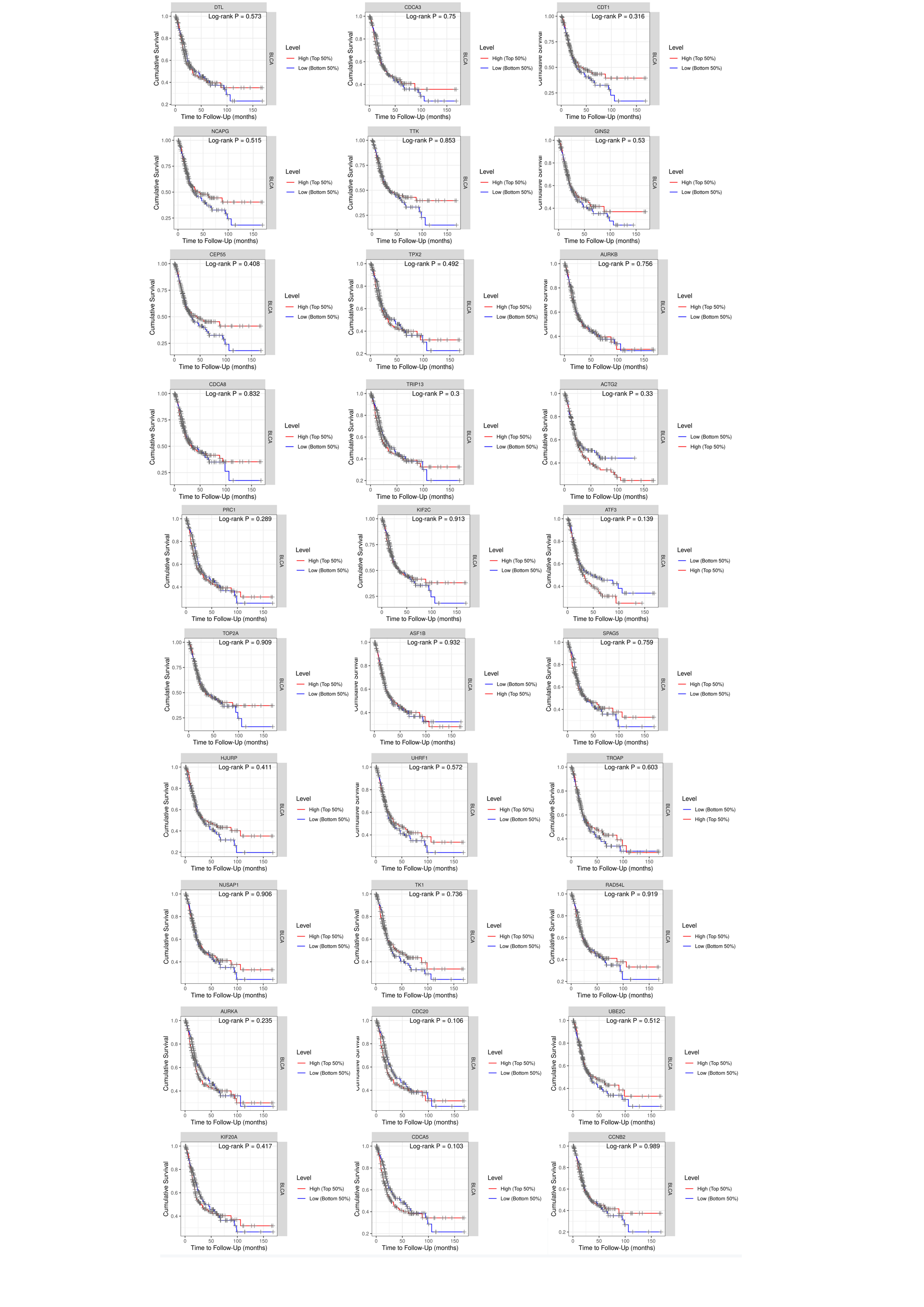

Supplement: Supplementary Figure 1 — Kaplan-Meier survival analysis the for 30 hub genes. [file Image_1.TIF]
